# Urinary Metabolomics of Plastic Manufacturing Workers: A Pilot Study

**DOI:** 10.3390/jox15020039

**Published:** 2025-03-04

**Authors:** Michele De Rosa, Ottavia Giampaoli, Adriano Patriarca, Federico Marini, Antonio Pietroiusti, Lorenzo Ippoliti, Agostino Paolino, Andrea Militello, Anna Rita Fetoni, Renata Sisto, Giovanna Tranfo, Mariangela Spagnoli, Fabio Sciubba

**Affiliations:** 1 Department of Chemistry, Sapienza University of Rome, 00185 Rome, Italy; michele.derosa@uniroma1.it (M.D.R.); adriano.patriarca@uniroma1.it (A.P.); federico.marini@uniroma1.it (F.M.); 2 NMR-Based Metabolomics Laboratory (NMLab), Sapienza University of Rome, 00185 Rome, Italy; ottavia.giampaoli@uniroma1.it (O.G.); fabio.sciubba@uniroma1.it (F.S.); 3 Department of Environmental Biology, Sapienza University of Rome, 00185 Rome, Italy; 4 Faculty of Medicine, Saint Camillus International University of Health Science, 00131 Rome, Italy; antonio.pietroiusti@unicamillus.org (A.P.); lorenzo.ippoliti@unicamillus.org (L.I.); 5 Department of Biomedicine and Prevention, University of Rome Tor Vergata, 00133 Rome, Italy; agostino.paolino@ptvonline.it; 6 Department of Medicine, Epidemiology, Environmental and Occupational Hygiene, Istituto Nazionale Assicurazione contro gli Infortuni sul Lavoro (INAIL), 00078 Monte Porzio Catone, Italy; a.militello@inail.it (A.M.); r.sisto@inail.it (R.S.); g.tranfo@inail.it (G.T.); 7 Department of Neuroscience, Reproductive and Odontostomatological Sciences-Audiology Section, University of Naples Federico II, 80131 Naples, Italy; annarita.fetoni@unina.it

**Keywords:** NMR-based metabolomics, occupational exposure, urinary profile, multivariate statistical analysis, plastic manufacturing

## Abstract

The plastic manufacturing industry has a crucial role in the global economy with a significant impact in a wide range of fields. The chemical risk to which workers are potentially exposed is difficult to characterize and strictly related to both the products and processes adopted. Among the chemicals used, we can cite styrene, phenol, butadiene and phthalates, but nano- and microplastic particles can also be released in the work environment. In this pilot study, we present for the first time an NMR-based metabolomic approach for assessing urinary profiles of workers employed in a plastic manufacturing company. Urine samples from twelve workers and thirteen healthy volunteers were collected and analyzed by NMR spectroscopy. Forty-six urinary metabolites belonging to different chemical classes were univocally identified and quantified. The dataset so obtained was then subjected to multivariate statistical analysis to characterize each profile and highlight any differences. An alteration in some metabolites involved in several pathways, such as amino acid metabolism and NAD metabolism, was found, and a strong impact on gut microflora was also speculated. Ultimately, our work has the objective of adding a tile to the knowledge of biological effects possibly related to occupational exposure even if it is below the threshold limit values.

## 1. Introduction

Nowadays, the Chemical Abstracts Service has univocally identified and cataloged approximately 219 million chemical substances (data updated to April 2024). In accordance with the European Regulation (EC) No. 1907/2006, for the registration, evaluation and authorization of chemicals, aimed to ensure a higher level of protection of human health, while not penalizing the European chemical industry; among all those cataloged, 26,865 different substances are in possession of marketing authorization. It is interesting to underline that the number of substances for which a professional exposure limit is foreseen, according to national legislation, is approximately 170, despite the more than 25 thousand present on the market as previously mentioned. These data suggest a condition of underestimation of occupational diseases caused by chemical agents, even in the case of full compliance with the current legislation. Metabolomics allows for early evaluation of occupational exposures, and it could be a useful tool that focuses attention on aspects that remain unclear today, such as the long-term effects at concentrations of toxics below the threshold limit values, or the evaluation of exposure to mixtures of substances or again exposures to both physical and chemical agents. The scope is to promote targeted risk assessments and develop personalized protective measures for the health of specific individuals or vulnerable groups, improving safety standards and potentially lowering occupational healthcare costs by focusing on prevention rather than treatment.

Plastic manufacturing represents one of the largest industrial sectors all over the world, with a critical role in the global economy and a significant impact in a wide range of applications such as packaging, construction, automotive and transportation, aerospace, electronics and even healthcare. According to a study by Grand View Research, the global plastic manufacturing industry was valued at over USD 700 billion in 2023 and is expected to grow in the next years. This market has surely experienced exponential growth over the past few decades [1], thanks to the increasing demand for plastic products, advancements in technology and low production costs. The entire production chain, from manufacturing and production to research and development, employs millions of people worldwide, and the Asia-Pacific region dominates the global market. Despite this, the industry is also facing important challenges, mainly related to the high environmental impact of plastic materials [2]. At the same time, from the point of view of occupational hygiene, the chemical risk correlated to this compartment is complex to characterize based on the wide range of products, subproducts and processes adopted. A detailed report published by the International Labour Organization [3] in 2023 indicates the potential risks to which workers employed in plastic manufacturing are exposed. Among this had to be included exposure to many chemicals such as hydrogen chloride, styrene, phenol, butadiene, formaldehyde, phthalates, lead, polybrominated diphenyl ethers (PBDEs) and perfluorinated substances (PFASs), used as additives or produced as by-products. These substances guarantee the desired modulation of material properties, and they are released mainly during the heating of raw materials for creating plastic products, in the form of fumes [4,5,6]. Moreover, a recent significant concern, not only from the occupational health point of view, is represented by microplastics. Microplastics (MPs) are defined as plastic particles with dimensions smaller than 5 mm [7] manufactured with the purpose of being added to other products (i.e., cosmetics, fertilizer, detergents) or accidentally created by the fragmentation and degradation of plastics. MPs exist in many different forms, including spheres, fibers or pellets, being present nowadays in all environmental matrices as ubiquitous pollutants [8]. These particles can also act as carriers for dangerous microorganisms or hazards present in the atmosphere [9,10]. More and more studies have shown potential risks for health related to MP exposure in terms of effects on the cardiovascular system, inflammatory lesions, metabolic disturbances, oxidative stress and even neurotoxicity [11,12,13]. Workers employed in the plastic manufacturing industry may be exposed to microplastic dusts, considering that exposure in workplaces can occur mainly through inhalation and ingestion. Nano- and microplastic particles (NMPs) can be generated during several activities related to the production of polymer beads or powder, or from processes such as extrusion, chopping, injection molding, 3D printing, utensil coating, laser cutting, high-speed drilling and treatment of polymer composites. Emission rates of NMPs would depend on the type of polymer and process employed [14,15].

In view of the above, the scenario appears very intricate, and consequently, the observable effects on the organism should be interpreted as a complex response to the global exposure conditions. In this regard, metabolomics, defined as “the quantitative measurement of the dynamic multiparametric metabolic response of living systems to pathophysiological stimuli or genetic modification” [16], has proven to be up to the task. The chosen analytical platform is the NMR spectroscopy because, as just demonstrated [17,18,19], it allows not only the simultaneous qualitative and quantitative analysis of hundreds of endogenous compounds in complex matrices but also a structural characterization and dosage of potentially unknown xenobiotic metabolites. It is important to note that to the best of our knowledge, this is the first study with the aim of investigating the urinary metabolic profile of workers employed in plastic manufacturing compartments.

## 2. Materials and Methods

### 2.1. Study Design

For this study, twelve workers employed in a plastic molding company and thirteen healthy volunteers as control group were enrolled. The characteristics of the subjects are reported in Table 1.

All samples were collected at the beginning of the working shift, after an overnight fasting, and in the middle of the working week. The control group consists of subjects enrolled on a voluntary basis, age- and sex-matched to the exposed subjects, whose occupation was known in order to verify the absence of occupational exposure to chemical agents of the same or different types compared to those examined. Each subject included in this study agreed to provide information on health status and main lifestyle habits. The absence of metabolic diseases, liver disease, diabetes, neoplastic pathologies, kidney disease or other pathologies that could seriously affect metabolism was checked. The absence of an impact of mean age differences between groups in relation to the main effect of exposure was also assessed (further details in Supplementary Figure S2).

All experiments were conducted according to the Declaration of Helsinki and followed the International Code of Ethics for Occupational Health Professionals, published by the International Committee of Occupational Health (ICOH). The information gathered was used as aggregate data referring to the whole group of workers, with no risk of individual identification. This study was approved by the Ethical Committee “Lazio 2”, study number: 31.23, protocol ID: 0041631 (no-profit study), 2 March 2023. Written informed consent was obtained from all the involved subjects.

### 2.2. Sample Preparation

All samples were prepared following a previously described protocol for biofluid metabolomics analysis [20] with some modifications. Briefly, an aliquot of 1200 µL of each sample was first added with 12 µL of NaN_3_ in order to obtain a bacteriostatic effect. Then, each sample was centrifuged at 4 °C for 15 min at 11,000 rpm to remove any cellular debris. Finally, 400 µL of the supernatant was added with 200 µL of buffer solution (PBS 200 mM, pH = 7) and with 60 µL of a solution of the internal standard 3-(trimethylsilyl) propionic-2,2,3,3-d_4_ acid sodium salt (TSP) in D_2_O for a final concentration of TSP in urine of 1.82 mM. All samples were stored at −80 °C until the NMR analysis.

### 2.3. 1H-NMR Spectroscopy for Urinary Metabolomics

NMR spectra were recorded according to [21]. In greater detail, all spectra were acquired with a JEOL JNM-ECZR spectrometer (JEOL Ltd., Tokyo, Japan), equipped with a magnet operating at 14.09 T and 600.17 MHz for the ^1^H frequency. For the acquisitions, the following setting parameters were employed: temperature of 298 K, 64 K points and 64 scans, spectral width at 9.03 kHz (15 ppm), presaturation pulse length of 2.00 s, relaxation delay of 5.72 s. All spectra were processed using ACD Labs software v.12.0 (Advanced Chemistry Development, Inc., 8 King Street East, Toronto, ON, Canada). After the multiplication for an exponential window function (LB = 0.3 Hz) and the application of Fourier Transform, spectra were manually phased and baseline corrected by applying the baseline correction FID reconstruction (BCFR) procedure. To allow compound identification, bidimensional experiments were also carried out on selected samples. Total Correlation Spectroscopy (TOCSY) ^1^H-^1^H experiments and Heteronuclear Single Quantum Coherence (HSQC) ^1^H-^13^C experiments were performed according to [22]. The assignment of the resonances (reported in Supplementary Table S1) was performed by the analysis of cross-correlated signals in 2D spectra and by comparison with the literature and open access databases [23,24]. For quantitative analysis, only signals free from overlapping have been chosen, manually integrated and normalized for the number of protons generating the signal. These values were then compared with the normalized integral of TSP (internal concentration standard), and the obtained concentrations were further normalized for creatinine concentration, referring to the singlet signal at 4.05 ppm. Quantities were finally expressed as μmol/mmol of creatinine (Supplementary Table S2).

### 2.4. Statistical Analysis

Firstly, Principal Component Analysis was applied to the dataset after autoscaling. Subsequently, with the aim of identifying variables significant for discriminating between two groups of healthy volunteers (CTRL) and exposed workers (Exposed), a Partial Least Square discriminant analysis (PLS-DA) model was built, choosing repeated double cross-validation (DCV) as the validation procedure as reported in other studies [25]. Model performance was evaluated by the following figures of merit: sensitivity, specificity, accuracy and percentage of correct classification. Finally, we considered only those variables whose sign along the first canonical variate (CV1) remained consistent during the cross-validation steps as significant [26].

From the univariate point of view, after evaluating the normality and homoscedasticity of the distribution for each variable, the Wilcoxon rank sum test or Student’s *t*-test was applied. Statistics were carried out employing MATLAB ver. R2023a equipped with the Statistics and Machine Learning Toolbox (Natick, MA, USA: The MathWorks Inc.) and in-house-written functions.

## 3. Results

**Figure 1:**
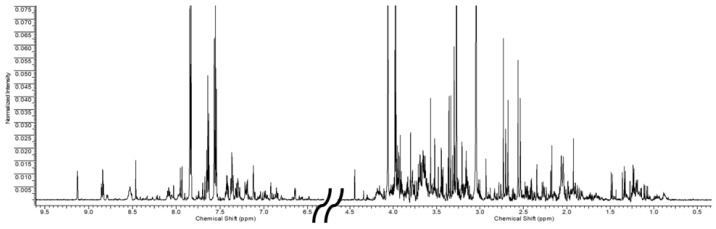
Representative ^1^H-NMR spectrum of human urine of both aromatic and aliphatic regiorns (to allow for better visualization, the portion of the spectrum containing the water and urea signals was removed).

Since no qualitative differences were found in urinary samples between the worker and healthy volunteer groups, a representative 1H-NMR spectrum is reported in Figure 1. Forty-six urinary metabolites, belonging to different chemical classes, were identified and quantified. In addition, four unknown compounds were also observed and quantified. The list of all quantified metabolites with the relative resonance chemical shifts is reported in Supplementary Table S1.

**Figure 2:**
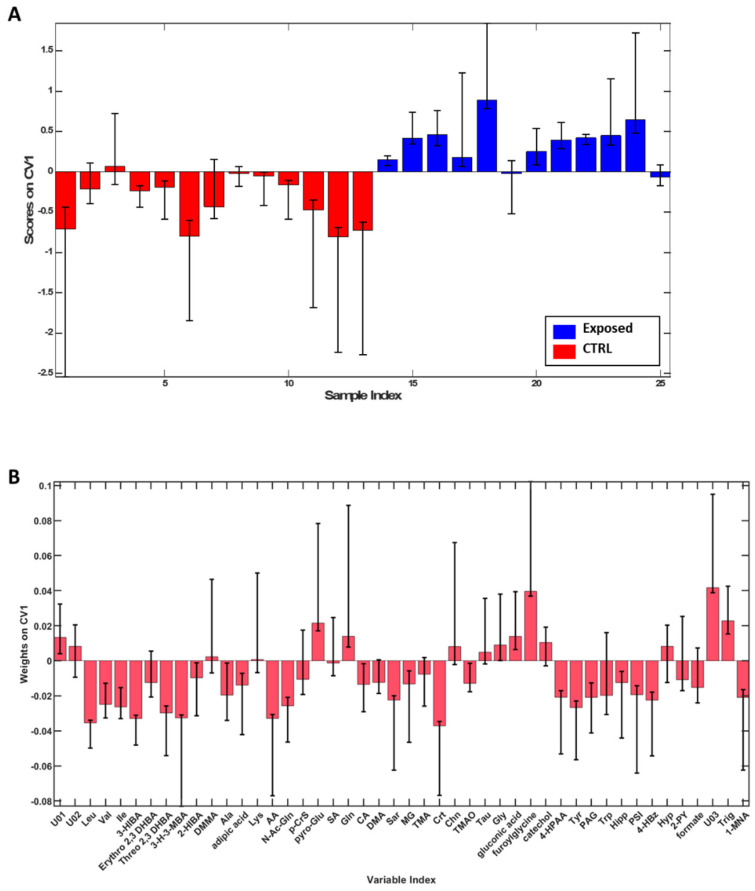
PLS-DA scores (**A**), weights on CV1 (**B**) plots for the comparison between the CTRL (red) and workers (blue). Only variables whose confidence interval bounds do not cross the threshold of 0 are considered significant. According to the described method, leucine (Leu), valine (Val), isoleucine (Ile), 3-hydroxyisobutyrate (3-HIBA), threo 2,3 dihydroxybutyrate (Threo 2,3 DHBA), 3-hydroxy-3-methylbutyrate (3-H-3MBA), alanine (Ala), acetate (AA), *N*-acetylglutamine (*N*-AcGln), citrate (CA), sarcosine (Sar), methyl-guanidine (MG), creatine (Crt), trimethylamine-*N*-oxide (TMAO), 4-hydroxyphenylacetate (4-HPAA), tyrosine (Tyr), phenylacetylglycine (PAG), hippurate (Hipp), pseudouridine (PSI), 4-hydroxybenzoate (4-HbzA), formate (FA) and 1-methylnicotinate (1-MNA) were found significant for CTRL, while pyro-Glutamate (pyro-Glu), glutamine (Gln), glycine (Gly), furoylglycine, trigonelline (Trig) and the unknown compound 3 (U03) were significant for the Exposed group.

To highlight spontaneous grouping or the presence of any outliers, Principal Component Analysis (PCA) was initially applied to the dataset, and it was possible to observe a tendency towards clustering based on exposure as shown in Supplementary Figure S1. Therefore, to better define the differences between groups, a supervised analysis, PLS-DA, was employed. The model built proved to be robust with an overall accuracy of 87.8 ± 4.1% and sensitivity and specificity of 85.5 ± 4.1% and 89.8 ± 6.1%, respectively, in distinguishing Exposed from CTRL. Then, with the aim to evaluate the contribution of each metabolite in discriminating two groups, variable weights along the first canonical variate were evaluated (Figure 2). According to the described method, leucine (Leu), valine (Val), isoleucine (Ile), 3-hydroxyisobutyrate (3-HIBA), threo 2,3 dihydroxybutyrate (Threo 2,3 DHBA), 3-hydroxy-3-methylbutyrate (3-H-3MBA), alanine (Ala), acetate (AA), *N*-acetylglutamine (*N*-AcGln), citrate (CA), sarcosine (Sar), methyl-guanidine (MG), creatine (Crt), trimethylamine-*N*-oxide (TMAO), 4-hydroxyphenylacetate (4-HPAA), tyrosine (Tyr), phenylacetylglycine (PAG), hippurate (Hipp), pseudouridine (PSI), 4-hydroxybenzoate (4-HbzA), formate (FA) and 1-methylnicotinate (1-MNA) were found significant for CTRL, while pyro-Glutamate (pyro-Glu), glutamine (Gln), glycine (Gly), furoylglycine, trigonelline (Trig) and the unknown compound 3 (U03) were significant for the Exposed group.

**Figure 3:**
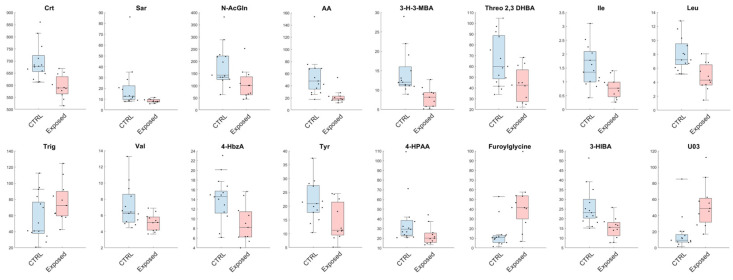
Metabolites found significant between healthy volunteers (CTRL) and exposed workers (Exposed) with a confidence level of 95% in the univariate statistical analysis. Details are reported in the Statistical Analysis section. The black dots represent the actual distribution of data for each variable. All data are expressed as µmol/mmol of urinary creatinine.

Analogously, from the univariate analysis, we have observed a reduction in urinary levels of Leu, Ile, Threo 2,3 DHB, 3-H-3-MBA, AA, *N*-AcGln, Sar, Crt, Val, 3-HIBA, 4-HPAA, Tyr and 4-HbzA in the Exposed group, with a contextual increase in concentration of furoylglycine and U03 in comparison to CTRL (Figure 3).

## 4. Discussion

As mentioned before, the complexity of exposure makes it impossible to untangle single effects related to specific xenobiotics; therefore, in our opinion, it is more correct to talk about a multiparametric response of an organism generated from adaptation to exposure. In this context, the first observation that stands out from the results shown in this work is a general decrease in the urinary concentration of branched-chain amino acids (Ile, Leu and Val) as well as of their principal catabolites 3-HIBA and 3-H-3MBA [27]. Beyond that, a similar trend was also observed in Ala and Tyr levels. Branched-chain amino acids (BCAAs) are essential amino acids whose catabolism, conversely to most others, does not take place in the liver due to low hepatic activity of branched-chain-amino-acid aminotransferase (BCAT), the first enzyme involved in their catabolic pathway, but in skeletal muscle [28]. BCAAs are principally involved in protein synthesis as well as energy production in addition to performing several important metabolic and signaling functions. Alanine is a non-essential amino acid derived from the reductive amination of pyruvate operated from the enzyme alanine transaminase. This molecule has a crucial role in energy metabolism and helps to maintain blood glucose levels during exercise or fasting but is also involved in ensuring an organism’s proper immune function and protein biosynthesis [29]. Tyrosine represents another example of a non-essential aromatic amino acid obtained starting from phenylalanine by the phenylalanine 4-hydroxylase complex [30,31]. Tyr is the precursor for catecholamine biosynthesis as well as for a few thyroid hormones and neurotransmitters. The ability to synthesize this compound was found to be depleted in patients with chronic kidney disease (CKD) [32,33]. Based on what has been stated, such a generalized response including a decrease in both essential and non-essential amino acids leads to different possible hypotheses regarding the causes of that variation. The first one considers this phenomenon as related to the influence of xenobiotics on renal amino acid transport systems. Effects of this kind of various substances were described before in the literature [34]. An alternative explanation could be related to an imbalance in nitrogen homeostasis. It is known for example that BCAAs are linked, via a series of transamination reactions, to the maintenance of nitrogen levels in the organism [35]. At the same time, a depletion of such metabolites at the peripheral level was found to reflect a deeper imbalance in nitrogen homeostasis in patients affected by chronic fatigue syndrome. On the other hand, is also possible to speculate about the involvement of gut microbiota. The host–microbiome relationship and its alteration following exposure to chemicals is a topic widely investigated [36,37,38,39]. It was shown, for instance, how halogenated compounds possess a negative impact on the gut microbiota, changing the Firmicutes to Bacteroidetes ratio to a dysbiotic one, or how polychlorinated biphenyls (PCBs) are capable of decreasing the gut richness, increasing pathogenic bacteria abundance and modulating bacterial metabolism in different animal models [39]. Last but not least, several examples were reported where MPs are considered as potential triggers of intestinal inflammation status and dysbiosis, resulting in a notable decrease in gut diversity in humans as well as in other species [40,41]. Regarding the results shown in this study, it was just demonstrated that the microbiota may modulate host amino acid availability through several mechanisms, including affecting intestinal protein digestion enzymes or modifying intestinal permeability [42,43,44], highlighting that changes in microbiome composition or metabolism could influence the host systemic amino acid pool [45,46,47]. To strengthen this last hypothesis presented, it should be noted that among the urinary metabolites considered significant in the discrimination between exposed and healthy volunteers, many were directly related to the intestinal microflora. In particular, we observed a generalized decrease in 2-HIB, acetate, PAG, hippurate, formate, 4-HPAA, 4-HBz, TMA and TMAO, all molecules included in bacterial pathways such as phenylalanine and tyrosine metabolism (PAG, AA and 4-HPAA), catabolism of dietary phenols (hippurate) and quaternary amines (TMA/TMAO) or anaerobic fermentation (formate) [48,49,50] and so all potentially influenced by the composition and activity of microbiota.

A completely opposite trend was observed for the amino acids glycine and glutamine, whose levels were found to be increased in workers. Glycine is the smallest proteinogenic amino acid, and although it can be endogenously produced, it was considered a conditionally essential amino acid [51] based on its crucial role in several biological functions and on the impact of its deficiency on health status in the long term. It was also suggested that glycine concentration represents the rate-limiting step in glutathione (GSH) synthesis [52]. Glutathione is a tripeptide involved in cellular defense mechanisms from oxidatively generated damage and in xenobiotic metabolism. Conjugation with GSH belongs to the phase II metabolic reactions operated by the CYP-450 complex through the GSH S-transferase enzyme [53]. Thus, high glycine levels could represent a biological strategy to maintain an adequate synthesis rate of glutathione employed for xenobiotic elimination.

Glutamine, an amino acid generated by glutamine synthetase (GS) through the condensation of glutamate and ammonia, is principally involved in urea synthesis, nitrogen clearance and gluconeogenesis [33]. Higher concentrations of this compound were also found in cases of occupational exposure to styrene vapors [54] and welding fumes [21], both related to the hyperactivity of the GS enzyme following potentially hepatotoxic xenobiotic exposure.

N1-methylnicotinamide (1-MNA) together with N1-methyl-2-pyridone-5-carboxamide (2-Py) and N1-methyl-4-pyridone-3-carboxamide (4-Py) are the major metabolites of NAD, and their urinary outputs are employed to investigate NAD turnover [55]. In this study, we did not measure concentrations of 2-Py and 4-Py because they were below the NMR limit of quantification; however, it could be interesting to highlight that we observed a similar trend in 1-MNA levels to that occurring in other occupational exposures [54]. In that case, an explanation was provided in terms of metabolic response for restoring the NAD+/NADH ratio as an effect of oxidative state imbalance in exposed workers, and the hypothesis was supported by a contextual change in oxidative stress biomarkers in urine. For this reason, we can speculate about a similar response to that just observed even if a deeper understanding of that mechanism through the analysis of such biomarkers could surely represent a valid perspective for future studies.

The last aspect we want to focus on is the significant increase (about 4 times), in the workers group, in the metabolite 2-furoylglycine. 2-Furoylglycine is a carboxamide obtained from the condensation reaction between the amino group of the amino acid glycine and 2-furoic acid that happens in the liver [56]. The role of this metabolite in urine is poorly investigated in the literature. Some studies associated 2-furoylglycine with changes in the gut microbiome or altered mitochondrial fatty acid beta-oxidation [57], but it also was proposed as a putative biomarker of coffee consumption [58]. However, in our opinion, the high level of this compound found in urine could be correlated with occupational exposure to 2-furoic acid. It is known that 2-furoic acid is largely used as a plasticizer, beyond being the main component in the production of furan resins to be used as casts in plastic molding plants [59]. Furfuryl alcohol, a wetting agent, a solvent for dyes and a corrosion inhibitor for fiber-reinforced plastics, and furfural, a rubber additive, are two further compounds widely used in plastic manufacturing and structurally related to 2-furoic acid, which could be rapidly converted in the liver [60]. The inhalation exposure to vapors of these compounds can happen during the thermal processing of plastic products, and it was demonstrated in the past how they were expelled predominantly as their glycine conjugate metabolites [61,62]. Regarding this last observation, we consider it could be interesting information obtained from our results, but surely, the purpose of 2-furoylglycine as a candidate new biomarker of exposure needs further studies with a greater sample size, which can also control sources of uncertainty about the nature of this metabolite such as diet or coffee consumption.

## 5. Conclusions

In this pilot study, for the first time, the urinary metabolic profiles of workers employed in plastic manufacturing were evaluated. The results found an altered amino acid metabolism after exposure, and a strong gut microbiota involvement was also hypothesized. An increment in urinary levels of Gln and Gly was found in common with other kinds of exposure suggesting active hepatic metabolism potentially related to xenobiotic elimination. The high concentration of 1-MNA was explained in terms of an imbalance in the NAD+/NADH ratio according to the literature data. Therefore, a novel potential role of 2-furoylglycine in correlation with 2-furoic acid exposure was also presented.

A limitation of this study is surely represented by the small number of subjects for each group, but this is strictly related to the principle of voluntary participation which requires individuals to consent to take part in the research, as well as to the specific characteristics of the Italian production structure, predominantly composed of small- and medium-sized enterprises. In addition to this, it is certainly worth underlining that it is impossible to completely eliminate the possible impact of confounding factors that could affect the metabolomic analysis, including both genetics and environmental elements such as lifestyle, diet or drugs. Despite this constraint, in our opinion, the present work contributes to addressing a significant gap in the existing literature on this topic. Our findings underscore the utility and applicability of the presented innovative approach by demonstrating the potential of the metabolomic platform in exploring unique and specific exposure scenarios. Future research endeavors could focus on expanding the sample size to improve the statistical power and robustness of the results. Additionally, subsequent studies may aim to integrate metabolomic data with findings obtained through other advanced analytical platforms, such as metagenomics, which could provide the information needed to validate the hypothesis of the real involvement of the intestinal microbiota in the metabolic variations observed following exposure. This would enable a more comprehensive and multidimensional understanding of urinary metabolic profiles, providing deeper insights into the complex interplay between exposure scenarios and their biological effects.

## Figures and Tables

**Table 1:** Characteristics of the investigated subjects.

	Age (Mean ± SD)	Gender (Male/Female)	Alcohol Intake (*n*)	Smoking (*n*)
Workers	45.2 ± 12.9	12/0	0	0
CTRL	55.9 ± 4.5	13/0	0	0

## Data Availability

The original contributions presented in this study are included in the article/Supplementary Materials; further inquiries can be directed to the corresponding author.

## Supplementary Materials

The following supporting information can be downloaded at https://www.mdpi.com/article/10.3390/jox15020039/s1: Figure S1: PCA analysis between CTRL and Worker groups; Figure S2: PCA analysis performed for age factor; Table S1: ^1^H-NMR resonance assignment; Table S2: Table of metabolite concentrations; Workplace Description. Reference [63] is cited in the supplementary materials
