# Supplementary material for: Urinary Metabolomics of Plastic Manufacturing Workers: A Pilot Study
Source: J Xenobiot. 2025 Mar 4;15(2):39. doi: 10.3390/jox15020039 (PMC11932285; doi:10.3390/jox15020039)
Supplement: Supplementary file 1 [file jox-15-00039-s001.zip › jox-3404876-Supplementary_round2.pdf]

Supplementary Materials

Urinary Metabolomics of Plastic Manufacturing Workers: A Pilot Study

Michele De Rosa, Ottavia Giampaoli, Adriano Patriarca, Federico Marini, Antonio Pietroiusti, Lorenzo Ippoliti, Agostino Paolino, Andrea Militello, Anna Rita Fetoni, Renata Sisto, Giovanna Tranfo, Mariangela Spagnoli and Fabio Sciubba

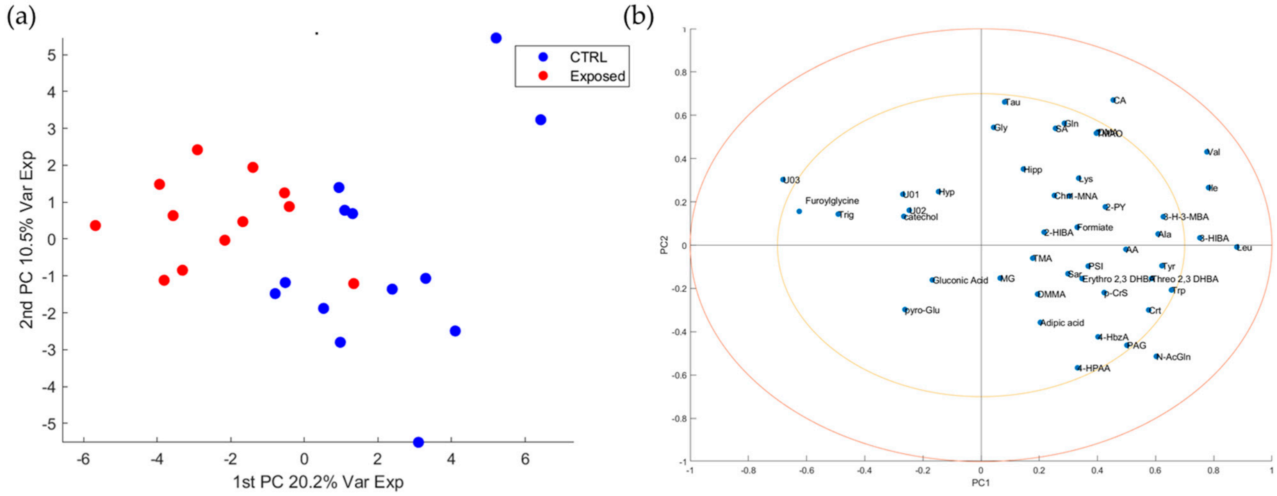

Figure S1. Score plot (a) and loading plot (b) obtained from PCA analysis carried out on the whole dataset as described in the text.

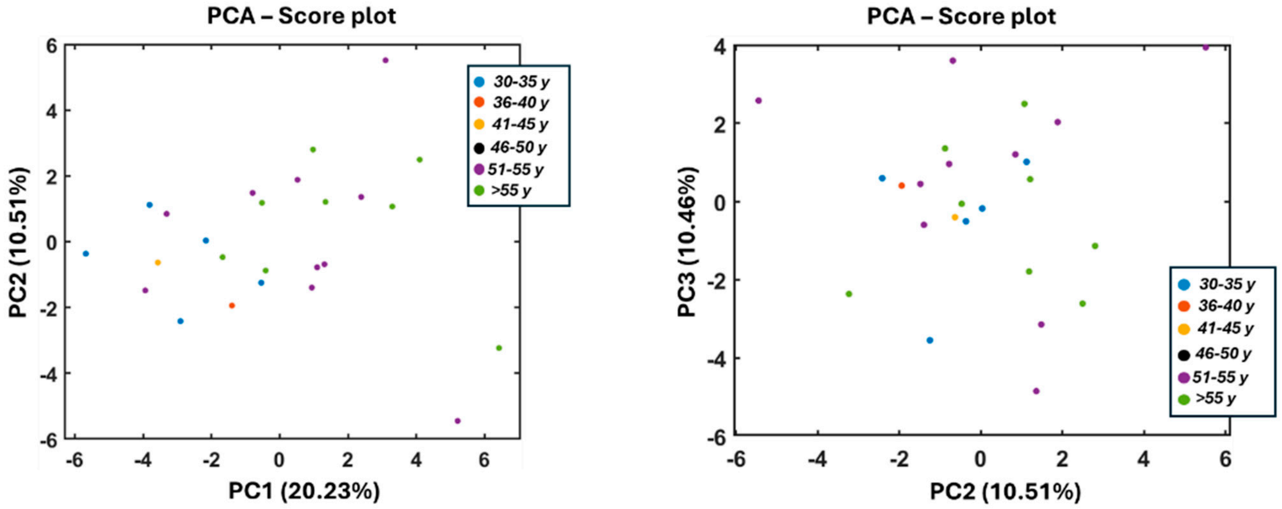

Figure S2. Score plots obtained from PCA analysis carried out on the whole dataset color coded for age factor. With the aim of verifying the presence of homogeneity in the dataset in relation to age, a PCA analysis was carried out by dividing the individuals based on age and therefore neglecting the exposure. As can be observed from the Score plots shown in the figure, there is no clustering of data based on the age of subjects, as was instead found for the exposure factor (Figure S1). On the basis of this analysis, it is possible to conclude that the age factor, although not totally

eliminable, is certainly of secondary importance and not comparable to the variability introduced by occupational exposure.

Table S1. All identified metabolites from 1H-NMR urinary spectra. * Identified but not quantified metabolites.

Metabolites	^{1}H δ ppm	Multiplicity	Assignment
1. Unknown compound 1 (U01)	**0.54**	d	
2. Unknown compound 2<br>U02	0.74	d	
3. Valine (Val)	0.99<br>**1.05**	d<br>**d**	CH3<br>**CH3′**
4. Leucine (Leu)	**0.95**<br>1.70<br>3.72	**t**<br>m<br>m	**CH3, CH3′**<br>CH2<br>CH
5. Isoleucine (Ile)	0.92<br>**1.01**<br>1.99	t<br>**d**	CH3<br>**CH3**<br>CH
6. 3-Hydroxyisobutyric acid (3-HIBA)	**1.07**<br>2.49<br>3.54<br>3.71	**d**	**CH3**
7. Erithro-2,3-dihydroxybutyric acid (Erythro-2,3-DHBA)	**1.11**<br>4.19	**d**<br>m	**CH3**<br>CH
8. Threo-2,3-hydroxybutyric acid (Threo-2,3-DHBA)	**1.13**<br>3.71<br>4.19	**d**<br>m<br>d	**CH3**<br>CH<br>CH
9. 3-hydroxy-3-methylbutirric acid (3-H-3-MB)	**1.27**	**s**	**CH3, CH3′**
10. Lisine (Lys)	1.48<br>**1.71**<br>1.89<br>3.02<br>3.74	m<br>**m**<br>m<br>m<br>m	CH2<br>**CH2**<br>CH2<br>CH2<br>CH
11. Lactate*	1.33<br>4.11	d<br>q	CH3<br>CH
12. Threonine (Thr)*	1.33<br>3.59<br>4.26	d<br>d<br>m	CH3<br>α-CH<br>β-CH
13. 2-hydroxyisobutyrric acid (2-HIB)	**1.36**	**s**	**CH3, CH3′**

14. Dimethylmalonic acid (DMMA)	**1.43**	**s**	**CH3, CH3′**
15. Alanine (Ala)	**1.49**<br>3.78	**d**<br>q	**CH3**<br>α-CH
16. Adipic acid	**1.54**<br>2.18	**m**<br>m	**α-CH2**<br>β-CH2
17. Acetic acid	**1.93**	**s**	**CH3**
18. N-acetylglutamine (N-AcGln)	1.95<br>2.12<br>**2.27**<br>4.18<br>7.97	 <br>**m**<br><br>bs	 <br>**CH2**
19. Pyroglutamic acid (pyro-Glu)	2.03<br>**2.40**<br>2.50<br>4.19	m<br>**m**<br>m<br>dd	CH2<br>**CH**<br>CH<br>CH
20. Succinic acid	**2.41**	**s**	α-CH2, β-CH2
21. Glutamine (Gln)	2.13<br>**2.46**<br>3.78	m<br>**m**<br>t	CH2<br>**CH2**<br>CH
22. p-Cresol Sulphate	**2.35**<br>7.21<br>7.28	**bs**<br>dd<br>dd	**CH3**<br>CH, CH′<br>CH, CH′
23. citric acid	**2.54**<br>2.69	**d**<br>d	**CH, CH′**<br>CH, CH′
24. Dimethylamine (DMA)	**2.73**	**s**	**CH3, CH3′**
25. Sarcosine (Sar)	**2.78**	**s**	**CH3**
26. Methylguanidine (MG)	**2.83**	**s**	**CH3**
27. Trimethylamine (TMA)	**2.89**	**s**	**CH3, CH3′, CH3′′**
28. Creatine (Crt)	**3.05**<br>3.95	**s**<br>s	**CH3**<br>CH2
29. Creatinine (Crtn)	3.03<br>**4.05**	s<br>**s**	CH3<br>**CH2**
30. Trimethtlamine N-Oxide (TMAO)	**3.27**	**s**	**CH3**<br>CH2
31. Taurine (Tau)	3.27<br>**3.43**	t<br>**t**	CH2<br>**CH2**
32. Glicine (Gly)	**3.57**	**s**	**CH2**
33. Gluconic acid	3.66<br>3.76<br>3.82	m<br>m<br>m	

	4.02<br>**4.12**	t<br>**d**	 **α-CH**
34. 2-Furoylglycine	3.94<br>**6.64**<br>7.19<br>7.70	d<br>**dd**<br>s<br>s	 **4-CH**
35. Catechol 1	**6.83**	**dd**	
36. 4-hydroxyphenylacetic acid(4-HPA)	**6.87**<br>7.17	**dd**<br>dd	**CH,CH**<br>CH,CH
37. Tirosine (Tyr)	**6.90**<br>7.18	**dd**<br>dd	**2-CH,6-CH**<br>3-CH,5-CH
38. 4-hydroxybenzoic acid (4-HBz)	6.97<br>**7.76**	dd<br>**dd**	2-CH,6-CH<br>**3-CH,5-CH**
39. Triptophane (Trp)	7.20<br>7.27-<br>7.29-<br>**7.50**<br>7.70	-<br>-<br>-<br>**pd**<br>pd	3-CH<br>2-CH<br><br>**5-CH**<br>4-CH
40. phenylacetilglycine (PAG)	**7.36**<br>7.42	**m**<br>m	
41. Hippuric acid (Hipp)	3.97<br>**7.55**<br>7.64<br>7.83	d<br>**m**<br>m<br>m	CH2<br>**3,5-CH**<br>4-CH<br>2,6-CH
42. Pseudouridine (PSI)	**7.67**	**bs**	**CH**
43. Hypoxanthine (Hyp)	**8.19**<br>8.21	s<br>s	2-CH<br>7-CH
44. N-Methyl-2-pyridone-5-carboxamide (2PY)	3.64<br>6.67<br>7.98<br>**8.33**	s<br>d<br>d<br>**dd**	N-CH3<br>3-CH<br>4-CH<br>**6-CH**
45. Formic acid	**8.46**	**s**	**CH**
46. Unknown compound 3 U03	8.06<br>8.54<br>**8.78**	 <br>**d**	 <br>**6-CH**
47. Trigonelline (Trig)	4.44<br>8.08<br>8.84<br>**9.12**	s<br>m<br>m<br>**s**	N-CH3<br>5-CH<br>4,6-CH<br>**2-CH**
48. 1-Methylnicotinamide (1-MNA)	8.17<br>8.89<br>8.96<br>**9.28**	t<br>d<br>d<br>**s**	5-CH<br>4-CH<br>6-CH<br>**2-CH**

Workplace Description

The work activity under study is carried out in the production department of a company inside an industrial shed where 20 different plastic hot molding machines are installed. In the same premises are the raw materials warehouse and a workshop for maintenance operations. The main activity is the hot molding of plastics (engineering polymers) using injection molding machines. The raw materials are in the form of granules and the hygroscopic ones are previously dried and dehumidified in special machines by circulating dry air. The medium used to generate dehumidified air consists of a bed of molecular sieves placed inside a dehumidifier. The dryer, on the other hand, consists of a hopper containing the material to be dried and a heating unit connected to it that has the function of generating hot air that is blown into the hopper. From the dryers, the materials are taken through automatic suction systems and, after a mixing phase, fed into the press hoppers. Shredded residues, recovered from previous processing, can be added to the material. The raw materials are then fed into the heated cylinder of the presses, where they are melted by the heat developed by electrical resistances; a worm screw homogenizes the plastic mass and conveys it to the cylinder head. The temperatures reached in the nozzle area can reach up to 380°C. Next, the injection of the melted plastic into the molds takes place, which is then cooled by means of a special cooling system that solidifies and maintains the shape acquired during injection. Subsequently, the parts removed from the molds are broken into individual elements, finished where necessary and assembled to obtain finished products for sale. The plastic molding work is carried out without interruption 24 hours a day in three shifts, five working days a week, for 11 months. Each press is controlled by a worker who intervenes in the event of a jam, for the addition of release agents to the molds and for maintenance of the press itself. A centralized and localized extraction system ensures air exchange through two stages of filtration (fabric and bag filters). It is believed that exposure to microplastics may occur at the same time as all work phases, including the dehumidification phase with hot air, the shredding of recovered parts from previous processes, the detachment from the molds and separation of individual elements and burr separation, the finishing phase, as well as from leaks or incomplete filtration of the dust suppression system, especially of the smallest particle size fractions. Particle formation and exposure to vapors can also reasonably be assumed during operations involving plastic melt due to high operating temperatures, such as during compression operations in molds. Some of the raw materials that can be used are listed below.

- press 32: PA6-GF30 (nylon, Fryanil)

- press 34: PA6-GF30 (nylon, Fryanil)

- press 31: PA66-GF20 (nylon, Latamid)

- press 35: PA6-GF30 (nylon, Fryanil)

- press 28: PPS-GF40 (polyparaphenylene sulfide, Ryton)

- press 26: PA66-GF25 (nylon, Latamid)

- press 27: PC-GF20 (polycarbonate, Latilon)

- press 25: POM (acetal resin, Hostaform)

- press 24: PEI-GF25 (PTFE, Ultem)
- press 22: PPS-GF65 ((polyphenylene sulfide, Larton)
- press 16: PC-GF10 (polycarbonate, Latilon)
- press 21: PPS-GF40 (polyparaphenylene sulfide, Ryton)
- press 30: PC-GF20 (polycarbonate)
- press 19: PC (Laprene)
- press 36: PA6 (Orgalloy)
- press 22: PPS-GF65
- press 16: PC-GF10
- press 33: PA66-GF20
- press 17: PA6-GF20
- press 18: PC

The acronym GF followed by the number indicates the presence of glass fibers and the relative percentage.

A detailed description od methods usable for the characterization of airborne ultrafine particulate matter in workplaces is provided in Boccuni et al., 2019 (F Boccuni, R Ferrante, F Tombolini, S Iavicoli, A Pelliccioni; Measurement of airborne ultrafine particles in work and life environments: study design and preliminary trends in an Italian university site.IOP Conf. Series: Materials Science and Engineering, 609 (2019) 042077 IOP Publishing; doi:10.1088/1757-899X/609/4/042077).
